# The Effect of Triptolide in Rheumatoid Arthritis: From Basic Research towards Clinical Translation

**DOI:** 10.3390/ijms19020376

**Published:** 2018-01-26

**Authors:** Danping Fan, Qingqing Guo, Jiawen Shen, Kang Zheng, Cheng Lu, Ge Zhang, Aiping Lu, Xiaojuan He

**Affiliations:** 1 Institute of Basic Research in Clinical Medicine, China Academy of Chinese Medical Sciences, Beijing 100700, China; fdp0406@gmail.com (D.F.); qingqingguo@hkbu.edu.hk (Q.G.); shenjiawen23@gmail.com (J.S.); zhengkang@hkbu.edu.hk (K.Z.); lcheng0816@gmail.com (C.L.); 2 Law Sau Fai Institute for Advancing Translational Medicine in Bone and Joint Diseases, School of Chinese Medicine, Hong Kong Baptist University, Kowloon Tong, Hong Kong; zhangge@hkbu.edu.hk (G.Z.); aipinglu@hkbu.edu.hk (A.L.); 3 School of Life Sciences and Engineering, Southwest Jiaotong University, Chengdu 610031, China; 4 School of Basic Medical Sciences, Shanghai University of Traditional Chinese Medicine, Shanghai 201203, China

**Keywords:** triptolide, rheumatoid arthritis, basic research, clinical translation

## Abstract

Triptolide (TP), a major extract of the herb *Tripterygium wilfordii* Hook F (TWHF), has been shown to exert potent pharmacological effects, especially an immunosuppressive effect in the treatment of rheumatoid arthritis (RA). However, its multiorgan toxicity prevents it from being widely used in clinical practice. Recently, several attempts are being performed to reduce TP toxicity. In this review, recent progress in the use of TP for RA, including its pharmacological effects and toxicity, is summarized. Meanwhile, strategies relying on chemical structural modifications, innovative delivery systems, and drug combinations to alleviate the disadvantages of TP are also reviewed. Furthermore, we also discuss the challenges and perspectives in their clinical translation.

## 1. Introduction

Rheumatoid arthritis (RA) is an immune-related disease that generally gives rise to continuous joint destruction, decreased expectancy of life and work ability, considerable disability, and even raised mortality [1]. Disease-modifying anti-rheumatic drugs (DMARDs), such as conventional synthetic DMARDs (csDMARDs) and biological DMARDs (bDMARDs), are currently the most commonly used drugs for treating RA. However, these drugs can not cure RA completely and often bring about severe side effects, such as infection and malignancies. Moreover, bDMARDs have low cost-effectiveness and bring a huge financial burden to the patients. Thus, it is still an imperative mission for researchers to find safer and more cost-effective medications.

**Figure 1:**
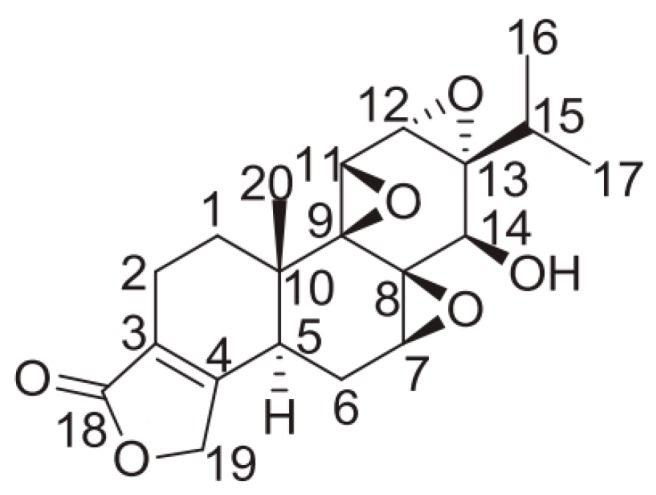
Chemical structure of (Triptolide)TP.

Traditional Chinese medicine (TCM), as an important kind of complementary and alternative medicine, is a precious resource for finding cost-efficient drugs, such as artemisinin. As for RA, there are many Chinese herbs with excellent immunosuppressive and anti-inflammatory functions [2]. *Tripterygium wilfordii* Hook F (TWHF) is a case in point. Tripterygium glycosides, extracted from TWHF, have been widely used to treat RA in China [3]. As the main active ingredient in Tripterygium glycosides, Triptolide (TP, a dierpene triepoxide in chemical structure, see Figure 1) has been considered as a promising anti-RA drug [4]. Increasing experimental evidence has verified its anti-RA effect. TP can significantly alleviate the severity of collagen-induced arthritis (CIA) in rats, with not only a potent anti-inflammatory effect but also the ability to prevent bone destruction [5,6]. Because of its outstanding anti-RA effect, TP has a great application potential in the clinic. Nonetheless, TP also exerts extreme toxicity and has poor water solubility, which impede its clinical application. Fortunately, many promising attempts for its clinical translation have been performed by researchers.

Thus, on the one hand, in order to gain a comprehensive and deep understanding of TP’s pharmacodynamic effect and toxicity in RA, related studies were summarized and reviewed in this paper; on the other hand, we also focused on the clinical translation researches of TP in RA hoping to get a better grasp of the progress in this area and provide proper directions and suggestions for its further study.

## 2. Effect and Mechanisms of Triptolide (TP) in Rheumatoid Arthritis (RA)

**Figure 2:**
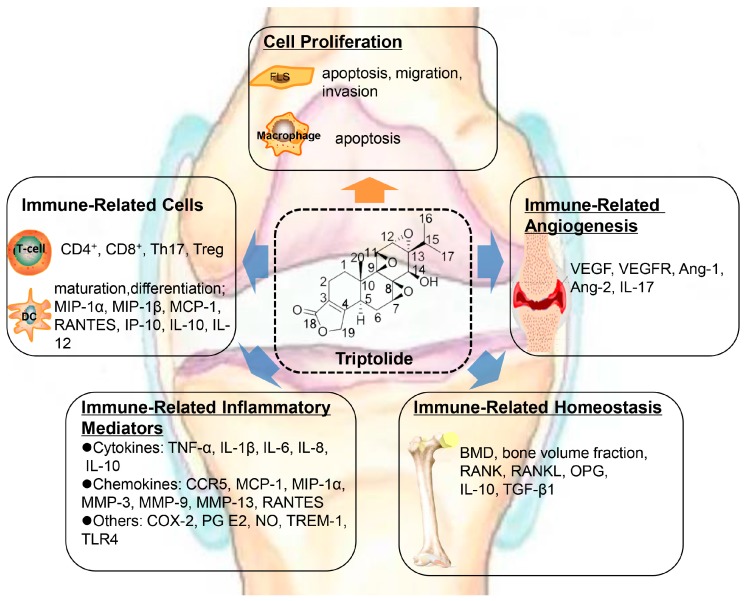
Schematic illustration of TP properties in the treatment of rheumatoid arthritis (RA). The anti-RA properties of TP have been attributed to its immunosupressive and antiproliferative effect. MIP: macrophage inflammatory protein; MCP: monocyte chemoattractant protein; RANTES: regulated upon activation normal T cell expressed and secreted; IP: interferon-induced protein; IL: interleukin; VEGF: vascular endothelial growth factor; VEGFR: vascular endothelial growth factor receptor; Ang: angiopoietin; TNF: tumor necrosis factor; CCR: C-C chemokine receptor; MMP: matrix metalloproteinase; COX: cyclooxygenase; PG: prostaglandin; NO: nitric oxide; TREM: triggering receptors expressed on myeloid cells-1; TLR: toll-like receptor; BMD: bone mineral density; RANK: receptor activator of nuclear factor-κB; RNAKL: receptor activator of nuclear factor-κB ligand; OPG: osteoprotegerin; TGF: transforming growth factor.

As a chronic immune-mediated inflammatory disease, immune regulatory factors play vital roles in the pathogenesis of RA. Until now, the anti-RA properties of TP in this condition have been attributed to its immunosupressive and antiproliferative effect (Figure 2).

### 2.1. Regulation of Immunological Functions

#### 2.1.1. Regulation of Immune-Related Cells 

T cells are among the key regulators of synovial inflammation in the development of RA, having both stimulatory and inhibitory roles [7] and playing a destructive or a protective role in bone metabolism in a context- and subtype-dependent manner [8]. TP was effective in preventing T cells proliferation [9]. CD4^+^ T cells play an important role in the induction and development of CIA, and CD8^+^ T cells might have a suppressive role in the etiology of CIA [10]. Previous studies showed that TP could increase CD8^+^ cells, while it decreased CD4^+^ cells in the Peyer’s patch. Therefore, the effect of TP on Peyer’s patch immune cells might partially explain some of the immunosuppressive activities of TP [11,12]. In addition, the overexpression of T cell receptor (TCR) variable gene (V gene) fragments can cause the activation and infiltration of autoreactive T cells. Nevertheless, TP was found to decrease the expression levels of TCR BV15 and TCR BV19. These changes might help explain the effectiveness of TP in the treatment of RA [13].

Th17 cells, a more recently characterized subset of CD4^+^ T cells, were shown to be more osteoclastogenic [8] and play an important role in the pathogenesis of RA through the production of Th17 signature cytokines [14]. Interleukin (IL)-6 and transforming growth factor (TGF)-β in mice or TGF-β and inflammatory cytokines in human are recognized as crucial factors necessary for the differentiation of naïve T cells into Th17 cells [14,15]. In vivo, TP significantly suppressed the production of Th17 cells from murine splenocytes and purified CD4^+^ T cells. Importantly, TP could inhibit the transcription of IL-17 mRNA and IL-6-induced phosphorylation of signal transducers and activators of transcription (STAT)3, which is a key signaling molecule involved in the development of Th17 cells. In vitro, TP reduced the production of collagen type II (CII)-specific IL-17 and the percentages of CII-specific IL-17^+^ CD4^+^ T cells in draining lymph nodes and spleens in CIA mice [16].

The dendritic cell (DC) is the most potent professional antigen-presenting cell (APC). Immature DCs (iDCs) have the ability to capture and process antigens in inflammatory tissues and undergo phenotypic and functional maturation implying the production of cytokines and chemokines in inflammatory microenvironments. Mature DCs produce multiple chemokines which act as chemoattractants for T cells, B cells, natural killer (NK) cells, and even neutrophils [17,18,19]. Therefore, DC is also regarded as an important target of immunosuppressants. Recently, research indicated that TP treatment inhibited lipopolysaccharide (LPS)-induced phenotypic changes and maturation of DCs [20,21]. TP also prevented the differentiation of immature human monocytes (MoDC) by inhibiting CD1a, CD40, CD80, and CD86 expression and upregulating CD14 expression [22]. In addition, the ability of DCs to stimulate allogeneic T cell responses was also impaired by TP. Furthermore, the production of IL-10 and IL-12 by DCs was modulated after TP treatment [20]. Yan et al. study indicated that TP might induce splenic DCs to CD11c^low^ differentiation, followed by shifting of Th1 to Th2 in vitro [23]. Cao et al. [24] conducted a study to investigate whether TP can inhibit DC-mediated chemoattraction of immune cells, because DC and chemokines are all important mediators in linking innate immunity and adaptive immunity. They found that TP impaired DC-mediated chemoattraction of neutrophils and T cells. Additionally, TP inhibited LPS-induced DC production of chemokines such as macrophage inflammatory protein (MIP)-1α, MIP-1β, monocyte chemoattractant protein (MCP)-1, regulated upon activation normal T cell expressed and secreted (RANTES), and interferon-induced protein 10 (IP-10) via suppression of nuclear factor kappa-light-chain-enhancer of activated B cells (NF-κB) activation and STAT3 phosphorylation. These data provided new insights into TP immunopharmacology.

#### 2.1.2. Regulation of Immune-Related Inflammatory Mediators

As RA is a complicated disease caused by a variety of factors, the inflammatory response has been considered as the main protracted cause of RA. The process of inflammation is usually tightly regulated by both mediators that initiate and maintain inflammation and mediators that shut the process down [25]. In states of chronic inflammation, an imbalance between the two types of mediators leaves inflammation unchecked, which leads to cellular damage. Previous studies have demonstrated that proinflammatory cytokines and chemokines produced by infiltrating immune cells and synoviocytes are implicated in the pathogenesis of RA. Meanwhile, plenty of cytokines and chemokines are also found in the synovial fluid of RA patients [26]. These cytokines and chemokines play an essential role in synovitis, pannus formation, and joint destruction caused by RA [27,28,29,30]. Previous studies showed that TP could lower the level of tumour necrosis factor (TNF)-α, IL-1β, IL-6, nuclear factor (NF)-κB, and cyclooxygenase (COX)-2 in ankle joints and serum in CIA rats [5,31]. Meanwhile, in LPS-induced mouse macrophages, TP could induce the reduction of toll-like receptor 4 (TLR4) proteins and of TIR-domain-containing adapter-inducing interferon-β (TRIF) adapter proteins in the MyD88-independent pathway of TLR4, confirming that both MyD88- and TRIF-mediated NF-κB activation might be suppressed by TP [32]. Moreover, TP decreased C-C chemokine receptor type 5 (CCR5) protein and mRNA levels in synovial tissue of adjuvant-induced arthritis (AIA) rats [33]. Except for CCR5, the overexpression of MCP-1, MIP-1α, and RANTES were also downregulated in TP-treated AIA rats [34]. Additionally, TP could inhibit prostaglandin (PG) E [2] production via a selective suppression of the production and gene expression of COX-2 in CIA rats [35]. Simultaneously, Wang et al. reported that TP could inhibit the production of nitric oxide (NO) by decreasing inducible NO synthase gene transcription [36]. Triggering receptor expressed on myeloid cells (TREM)-1 is a member of the Ig superfamily, and its activation can result in an inflammatory reaction [37,38]. We learned that the expression of TREM-1 could be activated by TLR through LPS, which could further lead to the production of proinflammatory cytokines via the NF-κB pathway [39,40]. Our study indicated that TP could significantly inhibit TREM-1 expressions in CIA rats, as well as decrease the production of TREM-1 in LPS-stimulated U937 cells, which demonstrated that TP could modulate the TREM-1 signaling pathway to inhibit the inflammatory response in RA [5]. TP suppressed TNF-α-induced expression of the IL-1β, IL-6, and IL-8 in fibroblast-like synoviocytes (FLSs) [41]. Treatment with TP also decreased the activation of matrix metalloproteinase (MMP)-3, MMP-9, MMP-13, and the cytoskeleton rearrangement of RA FLSs [42,43]. Moreover, TP not only decreased the IL-1α-induced production of proMMP-1 and 3, but also suppressed their messenger RNA (mRNA) levels in human RA FLSs. Conversely, the expression of tissue inhibitors of metalloproteinases (TIMPs) 1 and 2 induced by IL-1α was augmented by TP in the synovial cells [44]. In phorbol 12-myristate 13-acetate (PMA)-stimulated RA, the expression of IL-18 and IL-18 receptor (IL-18R) at protein and gene levels FLSs were also reduced by TP [45].

While some cytokines initiate and maintain the inflammatory process, others dampen it. The two best studied anti-inflammatory cytokines are IL-10 and IL-4. These cytokines cooperate to inhibit the production of inflammatory cytokines in vitro [46,47]. Xu et al. reported that TP could enhance the expression of IL-10 in regulatory T cells (Tregs) and further suppress osteoclast formation and bone resorption [6], and in vivo data revealed that the level of IL-10 was increased in the TP treatment group compared with the CIA group [13].

#### 2.1.3. Regulation of Immune-Related Angiogenesis

In the development of RA, blood vessel proliferation is common because of the influence of angiogenesis factors and angiogenic activators, like vascular endothelial growth factor (VEGF), fibroblast growth factor (FGF)-2, and hepatocyte growth factor in the inflamed and hypoxic environment. Angiogenesis is indispensable in perpetuating immune and inflammatory responses and can foster the infiltration of inflammatory cells into the joints, resulting in synovial hyperplasia and progressive bone destruction [48,49,50,51]. Previous studies suggested that TP could markedly reduce the capillary and the small, medium, and large vessel density in synovial membrane tissues of inflamed joints, and inhibit the expression of VEGF in the sera of CIA rats. The levels of VEGF, vascular endothelial growth factor receptor (VEGFR), Angiopoietin (Ang)-1, Ang-2, and IL-17 in the supernatants of human RA FLSs and human umbilical vein endothelial cells (HUVEC) were also decreased after TP treatment. These results implied that TP might possess an anti-angiogenic effect in RA both in vivo and in in vitro assay systems [52,53].

#### 2.1.4. Regulation of Immune-Related Bone Homeostasis

As an autoimmune disease characterized by inflammation and bone loss, bone homeostasis, which involves bone formation mediated by osteoblasts and bone resorption regulated by osteoclasts, is disrupted in the pathological condition of RA. The bone loss and joint destruction are mediated by immunological insults by various immune cells and inflammatory cytokines. The bone destruction that occurs in RA is also regulated by the receptor activator of nuclear factor-κB (RANK) and its ligand (RANKL), simultaneously [8]. Liu et al. found that TP could upregulate the bone mineral density (BMD), bone volume fraction, and trabecular thickness of inflamed joints and downregulate the trabecular separation, which suggests a protective role of TP on the volume and quality of the preserved trabecular bone despite joint inflammation [54]. Meanwhile, TP could significantly reduce the expression of RANKL and RANK, enhance the level of osteoprotegerin (OPG) in joints and sera of CIA rats, as well as decrease RANKL and RANK level and increase OPG production in the coculture system of human FLSs and peripheral blood mononuclear cells (PBMCs), which further revealed that TP might attenuate RA in part by preventing bone destruction, and inhibit osteoclast formation by regulating the RANKL–RANK–OPG signaling pathway [54]. Another study showed that the protective effects of TP on the joint destruction seen in RA might be associated with its inhibitory effect on the aggression of RA FLSs by blocking c-Jun N-terminal kinase (JNK) activation [42]. Furthermore, Tregs secrete cytokines like IL-10 and TGF-β1 that appear to play a key role in suppressing the differentiation of osteoclasts and the resorption of bone [55]. Research by Xu et al. indicated that TP could enhance the expression of IL-10 and TGF-β1 secreted by Tregs in vitro, which further inhibit osteoclast formation and bone resorption [6]. In another study, TP was found be able to reverse TNF-α-associated suppression of osteoblast differentiation, suggesting that TP might have a positive effect on bone remodeling [56].

### 2.2. Regulation of Cell Proliferation

Accumulating research suggests that FLSs contribute to synovial inflammation and joint destruction [57,58,59]. They play a crucial part in the initial stages of synovitis through the local production of proinflammatory cytokines and small-molecule mediators of inflammation [7,59]. TP could inhibit the proliferation of FLSs, arrest the cycle of FLSs, and induce apoptosis of FLSs [41,60]. In addition, the migration of FLSs to the cartilage and bone is regarded as a critical process in cartilage destruction in RA [59]. Yang et al. demonstrated that TP could suppress the migration and invasion of RA FLSs by partially blocking the phosphorylation of the JNK pathway [42]. 

Macrophages are found in the synovial membrane and are central effectors of synovitis. Macrophages act through the release of cytokines such as TNF-α and IL-1 [7]. TP treatment could result in macrophage apoptosis, while no obvious necrosis occurred [61]. The level of TNF-α in LPS-induced macrophages could be decreased by TP [62]. 

## 3. Mechanisms of TP Toxicity

Despite TP remarkable effect on RA, an increasing number of studies demonstrated that TP could induce toxicity, including hepatotoxicity, nephrotoxicity, reproductive toxicity, and so on. 

### 3.1. Hepatotoxicity

To evaluate the liver injury effect of TP, the serum activities of alanine transaminase (ALT), aspartate transaminase (AST), and lactic dehydrogenase (LDH) were used as biochemical markers. One study on C57BL/6 mice reported the time-dependent hepatotoxicity of TP, accompanied by an increasing trend of AST and ALT in the serum at 6 and 12 h, a peak at 24 h after TP (600 mg/kg) administration, and a decrease after 24 h [63]. Another study showed that ALT, AST, and LDH activities in serum were multiplied by 9.1, 9.8, and 3.0, respectively, which occurred in BALB/C mice treated only with TP (1.0 mg/kg) but not in control groups [64]. Additionally, the livers of TP-treated (0.5 mg/kg) mice showed hyperemic, mottled, fragile, and fuzzy structures, hepatocytes' nuclei displayed pyknosis and ruptures, and cytoplasmic staining was uneven with slight cell damage [65]. In contrast, after giving TP (0.1, 0.3 mg/kg) through intravenous administration once daily for 14 days, AST activity in the serum of Wistar rats significantly decreased as the TP dose increased, but there was no significant change in ALT [66]. Moreover, TP (200–400 μg/kg, 28 days) induced mitochondrial membrane depolarization in female Sprague Dawley (SD) rats, resulting in liver damage with microvesicular steatosis and hyperlactacidaemia, and was accompanied by an augmentation in reactive oxygen species (ROS) [67]. In addition, an abnormal immune response can induce organ or tissue damage influenced by CD4^+^ T cells such as Th17 and Tregs. Recently, Wang et al. reported that TP (500 μg/kg for 24 h) elevated the Th17/Treg ratio, which was positively correlated with ALT and AST in the serum, as well as acute liver injury of female C57BL/6 mice [63]. Recently, Yang and her colleagues found that the intragastric administration of TP (400 μg/kg body weight, 28 days) increased serum total bile acid and ALP levels and suppressed hepatic gluconeogenesis in Wistar rats, indicating that TP induced hepatotoxicity, and this hepatotoxicity was related to the sirtuin (Sirt1)/farnesoid X receptor (FXR) signaling pathway [68]. Simultaneously, Lu et al. suggested that TP could cause hepatotoxicity by reducing substrate affinity, activity, and expression of the CYP450 isoforms 3A, 2C9, 2C19, and 2E1 [69].

### 3.2. Nephrotoxicity

To estimate the nephrotoxicity of TP, blood urea nitrogen (BUN) and creatinine (Cr), which are important biochemical parameters in the serum, were used. Yang et al. reported that TP could cause a significant reduction of renal function characterized by a remarkable upregulation of Cr and BUN concentrations. Research about the relationship between TP-induced nephrotoxicity and oxidative stress indicated that TP caused serious oxidative stress after a single dose of 1 mg/kg in male SD rats, decreased the activities of renal superoxide dismutase (SOD) and glutathione (GSH), increased the level of malondialdehyde (MDA) and BUN, and caused structural damage [70]. In the meantime, TP induced severe damage in the renal structure, characterized by tubular epithelial cell detachment, necrosis, and tubular obstruction [71]. Furthermore, renal glomeruli were hyperemic, swelling, scattered, and necrotic after TP treatment [65].

### 3.3. Reproductive Toxicity

Except for hepatotoxicity and nephrotoxicity, toxicity for the reproductive system and an antifertility effect were also obvious. In female reproductive toxicity studies, TP caused prolonged estrous cycles and reduced the relative weights of the ovary and uterus [72]. In male reproductive toxicity studies, after treating with TP, the testis and epididymis weights were severely decreased. The cauda epididymis sperm content and motility even decreased to zero [73]. Studies have demonstrated that TP toxicity to the reproduction system emerged mainly through a disruption of the normal androgen and estrogen signaling [74]. Estrogen synthesis enzymes, aromatase and steroidogenic regulatory protein, play important roles in estradiol synthesis and estrogen signaling. TP could disrupt the expression of these three key proteins leading to estradiol synthesis reduction and reproductive dysfunction [75]. Intracellular ROS, glutathione peroxidase (GPx), and SOD are very important for testosterone generation. Studies found that TP had an influence on ROS, GPx, and SOD resulting in testosterone reduction. It was also found that TP could induce direct cytotoxicity in Leydig cells [76].

### 3.4. Further Toxicity

It is widely known that TP could cause reproductive toxicity, liver damage, and renal injury. However, TP could also lead to damage in other organs. TP acute poisoning could cause acute myocardial damage, such as myocardium swelling, denaturation, cytolysis, and contraction band necrosis. This toxicological effect of TP might be closely related to mitochondria and cell membrane functions [77]. Furthermore, there was also injury to the spleen after long-term TP administration. As an inflammation inhibitor, a long-time usage of TP could cause immunotoxicity in the spleen. Increased spleen index, spleen volume, and spleen weight could be seen in impaired spleens [66]. Gastrointestinal tract symptoms, such as nausea, anorexia, vomiting, diarrhea, gastrointestinal ulcers, and bleeding, were also a result of adverse reactions to TP [78]. In the meantime, TP could induce hematologic toxicity. In hepatic P450-deficient mice, the total number of platelets (PLT) and the number of white blood cells were reduced after TP treatment (0.5, 1.0 mg/kg). TP also decreased the absolute number and percent of lymphocytes, while it increased the absolute number and percent of neutrophils to a concentration of 1.0 mg/kg. There was no difference in the levels of red blood cells (RBC) or hemoglobin (Hb) after TP treatment [79]. Scientists confirmed that P450s was responsible for the metabolism of TP in the liver. P450s deficiency might cause an increase in the bioavailability and toxicity of TP [79]. In the study of Liu et al., TP (200 and 400 mg/kg/day for 28 days) showed a reduced toxicity and a higher metabolic rate in male SD rats linked to CYP3A2 which was the main metabolic isozyme in male rats, revealing the importance of CYP3A2 on the sex-based differences in TP toxicity [80]. Although there was no clear explanation of the effects of TP toxicity on RA, this research provided novel directions for further studies on TP toxicity.

## 4. Translational Research of TP

As mentioned above, the potent immunosuppressive and antiproliferative effects make TP a promising drug for clinical RA therapy. At the same time, its high toxicity as well as its poor water solubility greatly hinder TP’s clinical applications [73,81]. In order to improve the characteristics of TP, strategies relying on chemical structural modifications, innovative delivery systems, and drug combinations are increasingly employed by researchers [65,82,83].

### 4.1. Chemical Structural Modifications of TP

Many drugs like TP exert excellent therapeutic effects while simultaneously causing dramatic toxicity and displaying poor water solubility. Certain chemical properties of a compound can be changed by modifying its chemical structure. These modifications may be employed to increase water solubility or decrease the toxicity of a drug, thus making it available for clinical use. Over the past decades, several TP analogs (Table 1) have been developed and evaluated, mainly including (5R)-5-hydroxytriptolide (LLDT-8) [84], PG490-88 [85], LLDT-67 [86], LLDT-288 [87], and so on. Among these derivatives, LLDT-8 has comparable immunosuppressive and anti-inflammatory functions and a much lower toxicity compared to TP [88]. Its effects on RA have been proved by preclinical tests and Phase I clinical trials in RA patients [88,89]. With regard to its mechanism of action, LLDT-8 is thought to inhibit the activation of macrophages and regulate T cells proliferation and function [90,91].

### 4.2. Innovative Delivery System

Drugs with poor solubility in water have trouble dissolving in the gastrointestinal tract, engendering a low bioavailability. Some innovative delivery systems, like those obtained through nanotechnology and microemulsions, can be employed to enhance the delivery efficiency of medications [94,95]. Hence, studies of TP delivered by liposomes, nanoparticles, solid lipid nanoparticles, and microemulsions are summarized below and listed in Table 2.

#### 4.2.1. Liposomes

Chen et al. [96] developed a TP-loaded liposome hydrogel patch (TP-LHP) which was proved to improve the bioavailability of TP because of its stable and long-term release. Similar to TP, TP-LHP showed significant efficacy in CIA rats. Moreover, TP was delivered transdermally in this study, which can avoid the first-pass effects on the liver and abate gastrointestinal toxicity.

#### 4.2.2. Nanoparticles

Nanocarriers can reduce the side effects and increase the delivery efficiency of many drugs. Poly-γ-glutamic acid (γ-PGA) has been reported to be a promising drug carrier. Zhang and his colleagues created a nanodrug carrier system called γ-PGA-l-PAE-TP (PPT) by wrapping TP in a poly-γ-glutamicacid-grafted l-phenylalanine ethylester copolymer. PPT demonstrated controlled release behavior. This research indicated that PPT could alleviate free TP toxicity on murine macrophage RAW264.7 cells and normal C57/B6 mice. The nanodrug carrier system showed broad application prospects in RA treatment [97].

Poly(d,l-lacticacid) nanoparticles were used as TP carrier by Liu group. They fabricated TP-loaded poly(d,l-lacticacid) nanoparticles (TP-PLA-NPs) through the spontaneous emulsification solvent diffusion method with modifications. This delivery system caused TP to be burst-released initially and slow-released subsequently. In vivo tests demonstrated the significant inhibition effect of TP-PLA-NPs on AIA rats [98]. Furthermore, another study demonstrated that TP-PLA-NPs could effectively lower renal toxicity in rats [99].

#### 4.2.3. Solid Lipid Nanoparticles

Solid lipid nanoparticles (SLNs) were introduced as an innovative drug delivery system at the beginning of the 1990s. This system has become a promising alternative to liposomes, polymeric nanoparticles, and so on because of its merits, like nontoxicity, excellent biocompatibility, as well as large-scale production possibilities [106]. The solid matrix of SLNs can protect the loaded drug from degrading in the gastrointestinal tract [78,107]. SLNs can be employed in both topical application and oral administration. Mei et al. [100,101,102] found that SLNs could efficiently promote TP penetration into the skin. Furthermore, they also confirmed the anti-inflammatory effect of SLNs on carrageenan-induced rats as well as AIA rats, with improved safety and minimized toxicity compared to TP. Another research group compared the toxicokinetics and tissue distribution of TP-SLN versus free TP in rats, and the results suggested that TP-SLN enhanced TP absorption, with a slow release which may contribute to boost TP efficacy. Tissue distribution results showed that TP-SLN was more distributed in the lung and spleen than in plasma, liver, kidney, and testes. This explained why TP-SLN could mitigate the genital toxicity of TP [103].

#### 4.2.4. Microemulsions

Microemulsions are increasingly used for the transdermal delivery of drugs because of their several advantages, such as enhanced efficacy in transdermal applications over conventional formulations, elevated drug solubility, and ease of manufacturing [108]. A previous study prepared TP-loaded microemulsions and proved that they could penetrate in vitro through the mouse skin without obvious irritation to the skin [109]. Furthermore, Xu et al. [105] developed a kind of TP-loaded hydrogel-thickened microemulsion (TP-MTH) to treat RA through transdermal delivery. They testified its good effects without apparent local and systemic toxicities.

### 4.3. Drug Combinations

In the clinic, it has been found that drug combinations could be a good choice to solve drug toxicity. Drug combinations use several drugs that interact with multiple targets in the molecular networks of a disease and, in practice, may achieve better efficacy and lower toxicity than monotherapies. Thus, drug combinations can produce a synergistic effect without increased toxicity [110]. To solve TP toxicity, scientists have already found some drug, such as glycyrrhetinic acid and silymarin, which could produce a synergistic therapeutic effect, detoxication, or both.

#### 4.3.1. Glycyrrhetinic Acid

During the process of RA treatment, Licorice (*Glycyrrhiza glabra* L.) was often used combined with TWHF or TWHF preparations to reduce the latter’s adverse effects. Glycyrrhizin (GL) was considered a main active component of Licorice. Research showed that a combination of GL and TP could reduce the side effects of TP. The detoxifying effect of GL on TP was considered inseparable from GL’s selective influence on cytochrome P4503A (CYP3A). CYP3A, a major Phase I xenobiotic metabolizing enzyme, is responsible for regulating the metabolism of TP in the liver, avoiding the accumulation of TP [111]. By activating CYP3A, GL could accelerate the metabolism of TP and reduce the body exposure to TP. This suggested a significant protective action against chronic liver injury in rats [82]. In addition, many studies have reported that both GL and TP have an anti-inflammatory effect [112,113]. Furthermore, GL combined with TP produced a synergistic anti-inflammatory effect [114].

GL dissolves in water and transforms into glycyrrhetinic acid (GA), which is an important active ingredient with pharmacological properties [115]. Pharmacokinetic studies found that an extensive accumulation of TP in the liver caused liver damage [116]. This kind of liver damage could be reduced by the combination of GA and TP. The possible mechanism is that GA could reduce TP accumulation by promoting TP hepatic metabolic clearance. Several studies proved that GA could promote TP hepatic metabolic clearance, and this action was closely related to P-glycoprotein (P-gp) [117,118,119].

#### 4.3.2. Silymarin

The excessive release of inflammatory mediators could lead to immunological injury. TP combined with silymarin produced synergistic anti-inflammatory effects when treating inflammatory diseases like RA [35]. Silymarin is an active ingredient of *Silybum marianum* and it was reported to have various pharmacological functions. Silymarin was often used, alone or as a major component of various pharmaceutical preparations, as a hepatoprotective agent clinically. Additionally, silymarin has also exhibited protective effects against inflammation [120]. Short-term oral administration of silymarin exerted protective effects on TP-induced liver injury. The combination of silymarin and TP could produce a synergistic immunosuppressive effect by reducing the excessive expression of proinflammatory cytokines and inhibiting inflammatory signaling [121].

## 5. Discussion and Further Perspectives

Here, in this review, we examined the research on the pharmacodynamic effects, toxicity, and clinical translation of TP in RA. An increasing number of preclinical studies have testified the immunosuppressant, anti-inflammatory, and antiproliferative effects of TP which scientifically explain its good clinical effect on RA. Additionally, TP toxicity in RA is also increasingly studied. By analyzing a series of reports, we speculated that the potential primary effect of TP in RA might be achieved via its immunosuppressive property. As RA is a systemic disease, the effective and toxic mechanisms of TP in RA still need deep investigation. Perhaps, bioinformatic methods rising recently can be exploited to explore TP pharmacodynamics and toxicological mechanisms from a more systematic point of view.

In terms of the clinical translation of TP, several problems should be raised here. Firstly, we found that several derivatives of TP were synthesized and proven to possess effects comparable to those of TP and are even currently able to enter clinical trials. However, most of the derivatives are studied for cancer with only one of them used to treat RA. Furthermore, only LLDT-8 is still awaiting the outcomes of the clinical tests, although it showed promising anti-RA effects in preclinical studies. In addition, with regard to innovative delivery systems, targeted drug deliveries are becoming more and more popular because of their specific targeting of certain organs or cells. Nevertheless, the current targeted delivery systems of TP are mostly renal-targeted and tumor-targeted. For example, 3,5-dipentadecyloxybenzamidine hydrochloride (TRX-20)-modified liposomes [122], PF-A299–585 [123], 2-Glucosamine [124], and lysozyme [125] were reported to specifically deliver TP to the kidney. Carbonic anhydrase IX (CA IX) [126], AS1411 [127], and nanoformulations coated with folate [128] were used to specifically deliver TP to lung cancer, pancreatic cancer, and hepatocellular carcinoma cells, respectively. Investigations using targeted delivery system for TP to treat RA are still scarce. Thus, more research is needed to advance the application of TP in RA.

## Figures and Tables

**Table 1:** Main derivatives of TP.

No.	Compound Name	Chemical Structure	Modification Sites	Improved Characteristics Compared with TP	References
1	(5R)-5-hydroxytriptolide (LLDT-8)	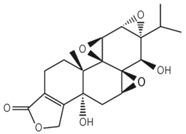	C-5 site	much lower toxicity	[88]
2	LLDT-67	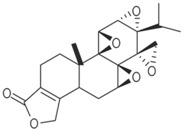	C-14 site	low toxicity	[86]
3	LLDT-288	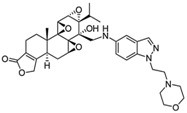	C-14 site	low toxicity	[87]
4	PG490-88	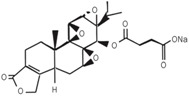	C-14-hydroxyl site	Water soluble	[85]
5	Minnelide	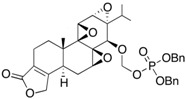	C-14-hydroxyl site	Water soluble	[92]
6	MRx102	——	——	low toxicity	[93]

Note: “——“means that there are no corresponding chemical structure and modification site in the literature we cited.

**Table 2:** Innovative delivery system studies of TP.

Drug Carrier	In Vivo/In Vitro	Advantages	References
liposome hydrogel patch	CIA rats	improves bioavailability of TP; bypasses hepatic first-pass metabolism, and reduces the incidence or severity of gastrointestinal reactions	[96]
nanodrug carrier system (γ-PGA-l-PAE-TP (PPT))	normal C57/B6 mice/RAW264.7 cell lines	reduces free TP toxicity in vitro and in vivo	[97]
poly(d,l-lactic acid) (PLA) nanoparticles	AIA rats	improve bioavailability of TP	[98]
poly(d,l-lactic acid) (PLA) nanoparticles	normal SD rats	abate the renal toxicity caused by TP	[99]
solid lipid nanoparticle hydrogel	carrageenan-induced rats	improves safety and minimizes the toxicity induced by TP	[100]
solid lipid nanoparticle/microemulsions	carrageenan-induced rats and AIA rats	increase therapeutic index	[101]
solid lipid nanoparticles	carrageenan-induced rats	enhance the anti-inflammatory activity of TP have a protective effect against TP-induced hepatotoxicity	[102]
solid lipid nanoparticles	normal SD rats	reduce gastric irritation	[78]
solid lipid nanoparticles	normal SD rats	enhance efficacy, decrease reproductive toxicity	[103]
nanostructured lipid carriers	normal SD rats	reduce subacute toxicity in male rats	[104]
hydrogel-thickened microemulsion	normal rabbits, mice, beagle dogs, guinea pigs	no obvious toxicities	[105]

Note: CIA: collagen-induced arthritis; AIA: adjuvant-induced arthritis; SD: Sprague Dawley; TP: triptolide.
